# Blind study evaluation illustrates utility of the Ion PGM™ system for use in human identity DNA typing

**DOI:** 10.3325/cmj.2015.56.218

**Published:** 2015-06

**Authors:** Jennifer D. Churchill, Joseph Chang, Jianye Ge, Narasimhan Rajagopalan, Sharon C. Wootton, Chien-Wei Chang, Robert Lagacé, Wenchi Liao, Jonathan L. King, Bruce Budowle

**Affiliations:** 1 Institute of Applied Genetics, Department of Molecular and Medical Genetics, University of North Texas Health Science Center, Fort Worth, TX, USA; 2 Human Identification, Thermo Fisher Scientific, South San Francisco, CA, USA; 3 Center of Excellence in Genomic Medicine Research (CEGMR), King Abdulaziz University, Jeddah, Saudi Arabia

## Abstract

**Aim:**

To perform a blind study to assess the capability of the Ion Personal Genome Machine™ (PGM) system to sequence forensically relevant genetic marker panels and to characterize unknown individuals for ancestry and possible relatedness.

**Methods:**

Twelve genomic samples were provided by a third party for blinded genetic analysis. For these 12 samples, the mitochondrial genome and three PGM™ panels containing human identity single nucleotide polymorphisms (SNPs), ancestry informative SNPs, and short tandem repeats (STRs) were sequenced on the PGM™ system and analyzed.

**Results:**

All four genetic systems were run and analyzed on the PGM™ system in a reasonably quick time frame. Completeness of genetic profiles, depth of coverage, strand balance, and allele balance were informative metrics that illustrated the quality and reliability of the data produced. SNP genotypes allowed for identification of sex, paternal lineage, and population ancestry. STR genotypes were shown to be in complete concordance with genotypes generated by standard capillary electrophoresis-based technologies. Variants in the mitochondrial genome data provided information on population background and maternal relationships.

**Conclusion:**

All results from analysis of the 12 genomic samples were consistent with sample information provided by the sample providers at the end of the blinded study. The relatively easy identification of intra-STR allele SNPs offered the potential for increased discrimination power. The promising nature of these results warrants full validation studies of this massively parallel sequencing technology and its further development for forensic data analysis.

The advent of massively parallel sequencing (MPS) technologies offers an alternative to current DNA typing methods. Comprehensive coverage of multiple forensically relevant genetic markers made possible by MPS technologies can provide a wealth of data for use in criminal investigations (1-6). While short tandem repeats (STRs) have been the primary marker system for human identity typing due to their polymorphic nature and high discrimination power, MPS allows for the examination of repeat and sequence variants in these STRs and for the inclusion of single nucleotide polymorphisms (SNPs) and mitochondrial DNA into the analysis pipeline. Using MPS to analyze STRs allows for the exact sequence of each allele to be obtained and for SNPs to be identified within the STR repeat structure. These intra-STR SNPs offer greater resolving power when analyzing mixtures and performing kinship analyses (1,4,7). Alternative marker types can facilitate analysis of degraded or low template samples. SNPs and mitochondrial DNA can aid in the analysis of degraded and low quantity samples. SNPs reflect a single base change, thus short amplicons can be used in their analysis (2,8). Sequencing the entire mitochondrial genome allows a greater discrimination power to be obtained and more accurate haplogroup assignments to be generated (3,5). This capability allows for better population background predictions and identification of maternal lineage relationships. The multitude of SNPs provides information on identity, ancestry, and lineage, which can help produce investigative leads that were not previously possible (6,8-11).

The Ion Torrent Personal Genome Machine® (PGM™) (Thermo Fisher Scientific, Waltham, MA USA) is one available MPS benchtop platform. The PGM™ is a high-throughput sequencer that employs semiconductor-sequencing technology (12). This sequence-by-synthesis chemistry measures the release of hydrogen ions as nucleotides are incorporated into the growing DNA strand. The PGM™ measures the associated, real-time pH change of the surrounding solution on a semiconductor chip thereby allowing for direct translation of chemically-encoded information into digital information (12). This process uses customized chemistries in a laboratory workflow that enables high-throughput and fast run times at a reasonable cost. In fact, the PGM™’s read length, sequencing time, running costs, and scalability lend itself to effective incorporation into diagnostic workflow of the forensic laboratory.

Twelve genomic samples were provided by a sample exchange group through the Green Mountain DNA Conference for a blinded genetic study. These samples were used to evaluate the potential of MPS on the PGM™ system to reliably analyze unknown samples, based on the self-declared information of the donors of these samples. The mitochondrial genome, identity SNPs, ancestry informative SNPs, and STRs (the latter three enabled by using PGM™ panels) were sequenced on the PGM™ system and resultant data were analyzed for these 12 samples.

## Materials and methods

### Samples

The study was conducted in June of 2014. Twelve blinded genomic DNA samples were kindly provided by George Duncan, Ron Fourney, and Bruce McCord for this study. The samples were obtained under informed consent from volunteers at the Broward Sheriff’s Office. Additionally, the policies and procedures approved by the Institutional Review Board for the University of North Texas Health Science Center in Fort Worth, TX were followed. These single-source samples arrived with arbitrarily assigned Sample Identification Numbers that were used to delineate the samples throughout the study. Extraction and quantification were completed by the sample providers using a Qiagen EZ1Advanced robot with a Qiagen DNA Investigator kit (Qiagen, Valencia, CA, USA) and a Promega Plexor HY real-time kit (Promega, Madison, WI, USA) on a 7500 Real Time instrument (Thermo Fisher Scientific), respectively. The concentrations of these samples ranged from 6.1 nanograms (ng)/microliter (μL) to 40 ng/μL, and a total volume of 7 μL per sample was provided.

### Capillary electrophoresis concordance data

Conventional STR typing was performed on the 12 genomic samples using the AmpFlSTR® Identifiler® Plus PCR Amplification Kit (Thermo Fisher Scientific) and the AmpFlSTR® Yfiler® PCR Amplification Kit (Thermo Fisher Scientific) and one nanogram of DNA for each reaction per the recommended manufacturer’s protocols (13,14). The GeneAmp® PCR System 9700 thermal cycler (Thermo Fisher Scientific) was used for PCR amplification. Electrophoresis was completed on an ABI Prism® 3100xl Genetic Analyzer (Thermo Fisher Scientific). Raw data were analyzed with GeneMapper® ID software v3.2.1 (Thermo Fisher Scientific).

### SNPs

*HID-Ion AmpliSeq™ Identity Panel and HID-Ion AmpliSeq™ Ancestry Panel*. The HID-Ion AmpliSeq*™* Identity Panel (Thermo Fisher Scientific) provides primers for amplification of 90 autosomal SNPs and 34 upper Y-clade SNPs (15). The average amplicon size for the autosomal SNPs is 132 base-pairs (bp), and the average amplicon size for the Y-SNPs is 141 bp (15). Additional information about this SNP panel can be found on Ion Community (*http://www.lifetechnologies.com/us/en/home/industrial/human-identification/next-gen-sequencing-for-forensics/hid-ion-ampliseq-identity-panel.html*). The HID-Ion AmpliSeq*™* Ancestry Panel (Thermo Fisher Scientific) contains primers for amplification of 165 autosomal SNPs with an average amplicon size of 130 bp (15). Additional information about this SNP panel can be found on Ion Community (*http://www.lifetechnologies.com/us/en/home/industrial/human-identification/next-gen-sequencing-for-forensics/hid-ion-ampliseq-ancestry-panel.html*).

*Library preparation*. Libraries for the two SNP panels were prepared and sequenced separately. One ng of input genomic DNA from each sample was used for library preparation. PCRs were completed using the Ion AmpliSeq*™* Library Kit 2.0 (Thermo Fisher Scientific), the HID-Ion AmpliSeq*™* Identity Panel or HID-Ion AmpliSeq*™* Ancestry Panel (Thermo Fisher Scientific), a GeneAmp® PCR System 9700 thermal cycler, and the manufacturer’s recommended protocol (15). The remainder of the library preparation process was completed following manufacturer’s recommended protocols (15). Final libraries for each sample were pooled in equal volume amounts, and 10 μL of the pooled library were used in the proceeding steps.

*Emulsion PCR and enrichment*. The pooled library was clonally amplified on Ion Sphere*™* Particles (ISPs) in an emulsion PCR. The emulsion PCR was completed using the Ion PGM*™* Template OneTouch*™* 2 200 Kit (Thermo Fisher Scientific) and the Ion OneTouch*™* 2 (Thermo Fisher Scientific) following the manufacturer’s recommended protocols (16). Emulsion PCR products were enriched for template-positive ISPs with the Ion OneTouch*™* Enrichment System (ES; Thermo Fisher Scientific) following manufacturer’s recommended protocols. The enrichment of the template-positive ISPs was assessed using the Ion Sphere*™* Quality Control Kit (Thermo Fisher Scientific) on the Qubit® 2.0 Fluorometer.

*Sequencing and data analysis.* Samples were sequenced on an Ion 318*™* v2 Chip (Thermo Fisher Scientific) with the PGM*™* system using the Ion PGM Sequencing 200 Kit*™* v2 (Thermo Fisher Scientific) and the manufacturer’s recommended protocols (17). Sequence data were analyzed using the Torrent Suite software v4.0.2 with the Alignment (v4.0-r77189), coverageAnalysis (V4.0-r77897), and HID_SNP_Genotyper (v4.1) plugins. The HID_SNP_Genotyper plugin makes SNP genotyping calls on PGM™ data using binary alignment map (BAM) files generated with the Torrent Suite software and BED files that specify the targeted areas of interest within the Hg19 reference genome. Integrative Genomic Viewer (IGV) was used for visualization of the aligned BAM files generated with the Torrent Suite software (18,19).

### STRs

*HID-Ion STR 10-plex Panel.* The HID-Ion STR 10-plex Panel (prototype from Thermo Fisher Scientific) contains primers for amplification of the amelogenin marker and nine STR markers [CSF1PO, D16S539, D3S1358, D5S818, D7S820, D8S1179, TH01, TPOX, and vWA]. Amplicon size for this panel ranges from 75-170 bp (1).

*Library preparation, emulsion PCR, and enrichment.* One ng of input genomic DNA from each sample was used for STR library preparation. PCRs were completed using the STR 10-plex PCR kit from Ion Torrent (Thermo Fisher Scientific), a GeneAmp® PCR System 9700 thermal cycler, and the manufacturer’s recommended protocol (20). The remainder of the library preparation process was completed following manufacturer’s recommended protocols (20). Final libraries for each sample were pooled in equal volume amounts, and 6 μL of the pooled library were used in the emulsion PCR. Emulsion PCR and enrichment of the amplified library were completed as previously described for the SNP libraries using an Ion PGM*™* Template OneTouch*™* 2 400 Kit (Thermo Fisher Scientific) instead of a 200 bp kit (21).

*Sequencing and data analysis.* Samples were sequenced and analyzed as previously described for the SNP libraries using an Ion PGM*™* Sequencing 400 Kit (Thermo Fisher Scientific) instead of a 200 bp kit and the HID_STR_Genotyper (v2.0) plugin vs the SNP specific plugin (22). Additionally, fastQ files generated with the Torrent Suite software were analyzed with the STR Allele Identification Tool – Razor (STRait Razor) (23,24) to verify the accuracy of the allele calls made by the HID_STR_Genotyper plugin.

### Whole mitochondrial genome

*Library preparation, emulsion PCR, and enrichment.* One ng of input genomic DNA from each sample was used for sequencing the entire mitochondrial genome. Library preparation was completed as described by Seo et al with the exception of the size selection step (25,26). The barcoded library was subjected to size-selection for fragments 400 bps in length using E-Gel® SizeSelect Agarose Gels (Thermo Fisher Scientific). The final size-selected and barcoded library was amplified using materials provided in the Ion Plus Fragment Library Kit (Thermo Fisher Scientific), 25 μL of library, 8 PCR cycles, a GeneAmp® PCR System 9700 thermal cycler, and recommended protocols (27). The amplified library was quantified and diluted to a concentration of 26 pM following manufacturer’s recommended protocols (28). Libraries for each sample were pooled in equal volume amounts. Ten μL of the pooled library were used in the emulsion PCR. Emulsion PCR and enrichment of the amplified library were completed as previously described for the SNP libraries using an Ion PGM*™* Template OneTouch*™* 2 400 Kit instead of a 200 bp kit (21).

*Sequencing and data analysis*. Samples were sequenced and analyzed as previously described for the SNP libraries with the following exceptions: an Ion PGM*™* Sequencing 400 Kit (22) was used; the variantCaller plugin (v4.0-r76860) replaced use of the HID_SNP_Genotyper plugin; and data were aligned to the revised Cambridge Reference Sequence (rCRS). Additionally, the variant call format (vcf) output files of the variant Caller plugin were used in mitoSAVE to generate haplotype calls in standard forensic conventions and output of a HaploGrep compatible file (29). HaploGrep (30) and EMMA (31) were used for haplogroup assignment. Circos plots were generated using Circos v0.64 (32).

### Final statistical analysis

Strand balance was calculated by dividing the coverage of one strand, showing lower coverage, by the coverage of the other strand, showing higher coverage, (eg, 450 × /500 ×  = 90%; 100% indicating equal coverage). Allele coverage ratios (ACRs) were calculated by dividing the coverage of one allele by the total coverage at that locus (eg, 450 × /970 ×  = 46%; 50% indicating equal coverage). Sequence coverage ratios used the bp sequence of the STR alleles vs the repeat structure of the alleles. Sequence coverage ratios were calculated by dividing the number of reads used to make nominal repeat length allele calls by the total number of reads at that marker. Genotypes from the Y-SNPs were used to identify manually each male’s Y-clade according to Y-DNA Haplogroup Tree 2013 (33) and provided population background information for the paternal side of their family.

## Results

### Library information

The sequencing runs for the Identity SNPs, Ancestry SNPs, STRs, and mitochondrial genome generated 330 Mbps, 349 Mbps, 247 Mbps, and 958 Mbps of sequence data, respectively. Taking into account a predicted error rate of one percent (Q20), the sequencing runs for the Identity SNPs, Ancestry SNPs, STRs, and mitochondrial genome produced 243 Mbps, 255 Mbps, 128 Mbps, and 798 Mbps of sequence data, respectively. The mean read length of the Identity SNPs, Ancestry SNPs, STRs, and mitochondrial genome libraries was 93 bps, 84 bps, 105 bps, and 201 bps, respectively.

### HID-Ion AmpliSeq*™* Identity Panel

**Figure 1:**
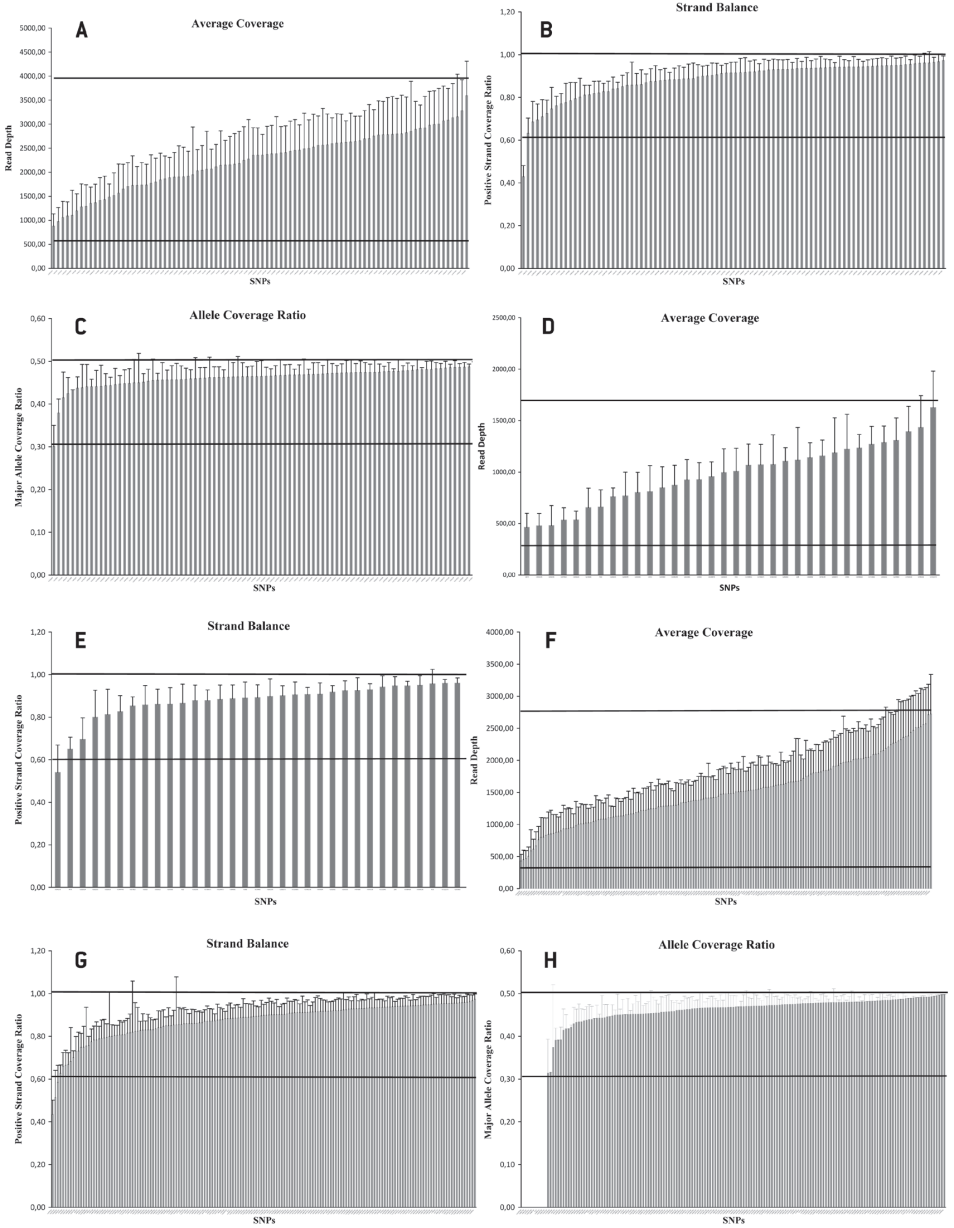
Average depth of coverage (**A**), strand balance (**B**), and allele coverage ratios (**C**) for the autosomal single nucleotide polymorphisms (SNPs) in the HID-Ion AmpliSeq*™* Identity Panel. Average depth of coverage (**D**) and strand balance (**E**) for the Y-SNPs in the HID-Ion AmpliSeq*™* Identity Panel. Average depth of coverage (**F**), strand balance (**G**), and allele coverage ratios (**H**) for the SNPs in the HID-Ion AmpliSeq*™* Ancestry Panel. Horizontal bars in the Average Coverage graphs represent two standard deviations from the mean.

The HID-Ion AmpliSeq*™* Identity Panel enables typing of 90 autosomal SNPs and 34 upper Y-clade SNPs. Genotypes were generated for all 124 Identity SNPs on all male samples and for all 90 autosomal Identity SNPs on all female samples. The performance of the autosomal SNPs and Y-SNPs in the Identity Panel was analyzed separately. The average depth of coverage across the 90 autosomal SNPs in the Identity panel was 2233 × . The coverage for each marker fell within two standard deviations of the mean (569 × -3898 × ), indicated by the horizontal bars in Figure 1A. Eighty-nine of the 90 (99%) autosomal SNPs fell within the 60%-100% strand balance range (Figure 1B). One-hundred percent of the autosomal SNPs had an ACR of 30%-50% (Figure 1C). The Y-SNPs yielded an average depth of coverage of 975 × . The coverage for each marker fell within two standard deviation of the mean (272 × -1678 × ), indicated by the horizontal bars in Figure 1D. Thirty-three of the 34 (97%) Y-SNPs fell within the 60%-100% strand balance range (Figure 1E).

In addition to evaluating the overall performance of the Identity panel, genotypes from the Identity SNPs were used to provide information about each of the 12 samples analyzed. The Y-SNPs, in particular, are useful for identifying sex, familial relationships, and paternal lineage. Out of the 12 samples analyzed, 5 donors were identified as male and 7 donors as female (Table 1). Differences in the Y-SNP haplotypes among the males indicated that there was no evidence of paternal relationships among them (Table 2). The Y-SNP data also were used to identify each male’s Y-clade and provide paternal lineage population affinity information (Table 1).

**Table 1:** Sex identifications, bioancestry, and haplogroup assignments generated in blind study

Sample	Sex*	Y-clade/region^†^	Biogeographic ancestry from ancestry informative markers (AIMs)^‡^	MitochondrialDNA (mtDNA) haplogroup^§^
1	Male	R1b/West Asia, Russian Plain, or Central Asia	European	J1c5
3	Female	-	European	H3b
4	Male	Q/Central Asia, the Indian Subcontinent, Siberia	Asian	U7b
5	Female	-	European	H6a1b4
6	Male	J/Arabian Peninsula	European	H33
7	Male	O2/Asia	Asian	M7b1a1c1
10	Female	-	European	H5n
13	Female	-	African American	L2a1f
14	Female	-	African admix	H1c
15	Female	-	African admix	H1c
16	Male	E/Africa	African	L3e1a1a
17	Female	-	European	H1c

*Sex identifications made based on presence or absence of Y-single nucleotide polymorphisms (SNPs).

†Bioancestry assignments based on Y-SNPs in the Identity Panel.

‡Population assignments based on SNPs in Ancestry Panel.

§Haplogroup assignments and maternal lineage with mtDNA.

**Table 2:** Y-single nucleotide polymorphisms (SNPs) results for the 5 male samples

Sample	rs2534636	rs35284970	rs9786184	rs9786139	rs16981290	rs17250845	L298	P256	P202	rs17306671	rs4141886	rs2032595	rs2032599	rs20320	rs2032602	rs8179021	rs2032624	rs2032636	rs9341278	rs2032658	rs2319818	rs17269816	rs17222573	M479	rs3848982	rs3900	rs3911	rs2032631	rs2032673	rs2032652	rs16980426	rs13447443	rs17842518	rs2033003
1	C	C	A	G	C	G	T	G	T	T	A	T	T	G	T	C	C	G	G	G	G	C	A	C	C	G	A	A	T	T	T	A	G	C
4	C	C	C	G	C	G	T	G	T	T	A	T	T	G	T	T	A	G	G	A	G	C	A	C	C	G	A	A	T	T	T	A	G	C
6	C	C	C	G	C	C	T	G	T	A	A	T	T	G	T	C	A	G	G	A	G	C	A	C	C	C	A	G	T	T	T	A	G	A
7	C	C	C	G	A	G	T	G	T	T	A	T	T	G	T	C	A	G	G	A	G	C	A	C	C	G	A	G	T	T	G	G	G	C
16	C	C	C	A	C	G	T	G	T	T	G	T	T	G	T	C	A	G	G	A	G	C	A	C	T	C	A	G	T	C	T	A	T	A

### HID-Ion AmpliSeq*™* Ancestry Panel

**Figure 2:**
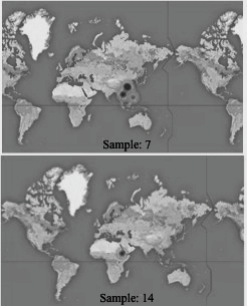
Example of output from HID_SNP_Genotyper plugin for ancestry predictions.

The HID-Ion AmpliSeq*™* Ancestry Panel enables typing of 165 autosomal SNPs. Genotypes were generated for all 165 SNPs on all 12 samples. The average depth of coverage across the 165 SNPs in the Ancestry Panel was 1511 × . The coverage for each marker fell within two standard deviations of the mean (240 × -2783 × ), indicated by the horizontal bars in Figure 1F. One-hundred sixty-two of the 165 (98%) SNPs fell within the 60%-100% strand balance range (Figure 1G). One-hundred fifty-five of the 165 (94%) SNPs had an ACR of 30%-50% (Figure 1H). The remaining 10 SNPs were homozygotes for all individuals. The HID_SNP_Genotyper plugin outputs ancestry results in the form of a heatmap of predicted likelihood of a person belonging to a particular population overlaid on a world map (Figure 2). Genotype data from the SNPs in the Ancestry Panel were used to predict the general population background of each individual (Table 1).

### HID-Ion STR 10-plex Panel

**Figure 3:**
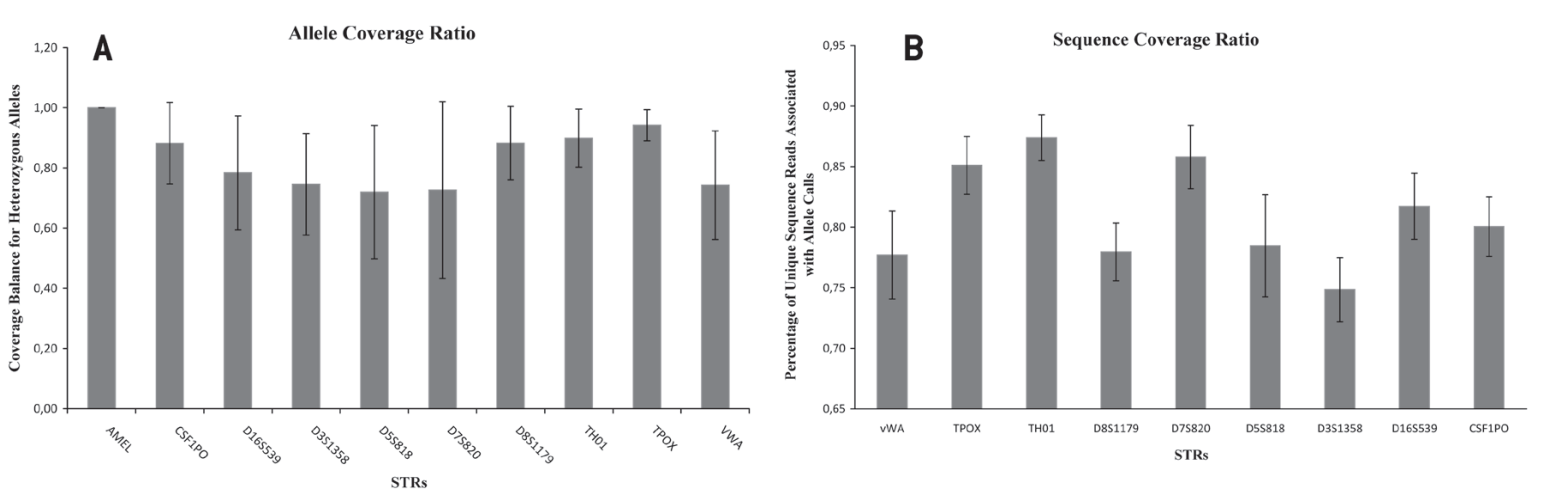
Allele coverage ratios (**A**) and sequence coverage ratios (**B**) for the markers in the HID-Ion STR 10-plex panel. Error bars represent standard deviation.

The HID-Ion STR 10-plex Panel contains primers for amplification of Amelogenin and 9 STR markers. Genotypes were generated for all 10 markers on all 12 samples (Table 3). Genotype calls produced by the HID_STR_Genotyper plugin and STRait Razor were completely concordant. Additionally, STR genotypes generated by MPS were concordant with data generated by capillary electrophoresis (CE) analysis. ACRs for the STRs ranged from 70%-100% (Figure 3A). Sequence coverage ratios allow for examination of the percentage of unique sequence reads associated with allele calls vs the unique sequence reads associated with stutter, noise, or PCR/sequencing errors. Sequence coverage ratios for STR markers ranged from 75%-90% (Figure 3B). Finally, sequence variants were identified among the alleles of 3 STR markers (D3S1358; D8S1179; vWA) analyzed in this study. Two different variants each were observed for alleles 14, 15, and 16 of the vWA locus, and 3 variants each were observed for alleles 15 and 16 of the D3S1358 locus. For alleles 12, 13, and 14 of the D8S1179 locus there were 2, 3, and 2 varying sequences, respectively. As an example of the variation, the 3 varying sequences seen for the 13 allele for the D8S1179 marker are listed in Table 4.

**Table 3:** Short tandem repeat (STR) profiles of the 12 samples sequenced in this study

Sample	AMEL	CSF1PO		D16S539	D3S1358	D5S818	D7S820	D8S1179	TH01	TPOX	vWA
1	X,Y	11,11	8,12		16*,17	12,12	8,10	13,13	8,9.3	10,12	17,18
3	X,X	10,11	12,12		15,18	9,11	11,12	12*,13	9.3,9.3	11,11	16,17
4	X,Y	12,15	12,13		16*,17	10,12	10,10	11,15	9,9.3	8,8	16,17
5	X,X	11,11	10,12		15,15	11,12	10,11	12*,13	6,7	8,8	15,19
6	X,Y	11,12	10,11		15,17	11,13	8,11	12*,13	7,9.3	8,11	16*,17
7	X,Y	10,12	9,12		15*,16	9,10	11,11	12*,13	6,9	10,11	14,18
10	X,X	12,12	11,14		16,18	11,11	10,11	11,13	7,9	9,11	14*,15
13	X,X	12,12	11,12		16*,17	12,13	10,11	14*,14*	6,7	9,11	15,18
14	X,X	12,13	9,12		16*,17	12,12	8,8	13*,13*	6,8	8,9	15*,17
15	X,X	12,12	9,13		16,17	11,12	8,9	10,13	6,9	9,9	15,16
16	X,Y	11,13	9,9		15,17	12,13	8,11	12,13	6,8	8,11	15,17
17	X,X	10,12	11,12		15*,16	12,13	8,8	13*,13*	7,8	8,9	17,19

*Indicates the presence of a sequence variant in this allele within or among individuals.

**Table 4:** An example of the sequence variations observed among one allele in the short tandem repeat (STR) marker D8S1179

Sample	Locus	Length	Allele	Counts	Sequence
1	D8S1179	52	13	7548	TATCTATCT**G**TCT**A**TCTATCTATCTATCTATCTATCTATCTATCTATCTATC
14	D8S1179	52	13	7232	TATCTATCT**A**TCT**G**TCTATCTATCTATCTATCTATCTATCTATCTATCTATC
14	D8S1179	52	13	6072	TATCTATCT**G**TCT**A**TCTATCTATCTATCTATCTATCTATCTATCTATCTATC
17	D8S1179	52	13	3906	TATCTATCT**A**TCT**A**TCTATCTATCTATCTATCTATCTATCTATCTATCTATC
17	D8S1179	52	13	3179	TATCTATCT**G**TCT**A**TCTATCTATCTATCTATCTATCTATCTATCTATCTATC

### Whole mitochondrial genome sequencing

**Figure 4:**
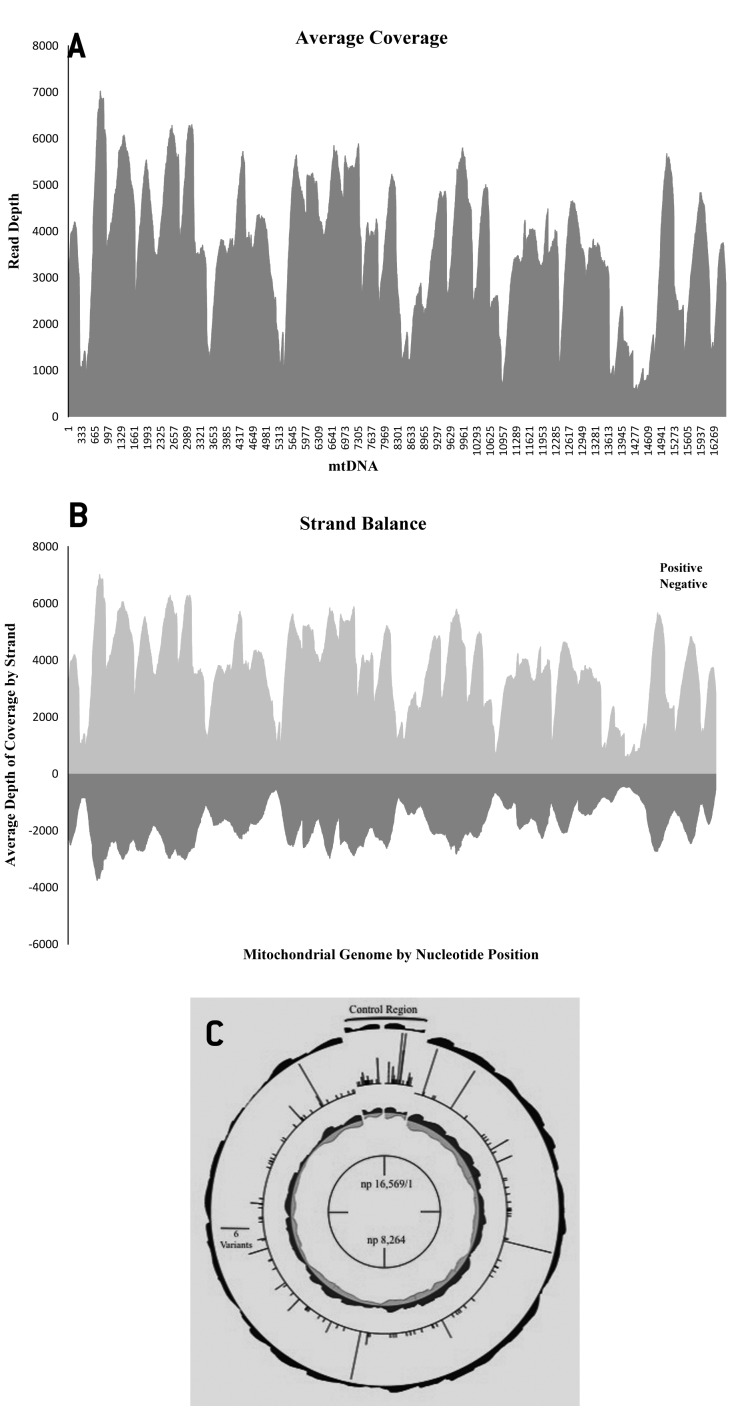
Average depth of coverage across the entire mitochondrial genome (**A**), strand balance across the entire mitochondrial genome (**B**), and a circos plot showing average coverage (outer layer), total number of identified variants (middle layer), and strand balance (inner layer; darker color showing positive strand coverage) across the entire mitochondrial genome for the 12 samples analyzed (**C**).

The mitochondrial genome was sequenced for the 12 individuals included in this study, and depth of coverage across the entire mitochondrial genome ranged from 489-7029 × (Figure 4A). Coverage plots from IGV showed areas of consistently high and low coverage across all individuals similar to that observed by Seo et al and King et al (3,26). Strand balance across the mitochondrial genome ranged from 30%-86%, a range also similar to that observed by Seo et al and King et al (Figure 4B) (3,26). Since mitochondrial DNA is a circular genome, a Circos plot was created to illustrate the depth of coverage, sequence variants identified, and strand balance of the mitochondrial genome sequence data (Figure 4C). A minimum coverage threshold of 10 × was set for mitochondrial DNA variant calls. These variant calls were output in the form of a vcf file and used to generate haplotype calls. Haplotype calls generated by mitoSAVE were input into HaploGrep and EMMA for haplogroup assignments (Table 1). Both analysis methods produced concordant haplogroup assignments. Three of the 12 individuals had the same haplotype (and thus haplogroup; ie, haplogroup H1c), suggesting a potential maternal relationship among them (Table 1).

### Identifying familial relationships

Previously discussed genotype data from the Identity SNPs and STRs allowed for expansion and refinement of relationships indicated by mitochondrial DNA sequence data. Sample 14 was found to be the mother of sample 15 and the daughter of samples 16 and 17. Likelihood ratios were generated for this pedigree (true pedigree vs four unrelated individuals) with in-house Thermo Fisher Scientific software. STR profiles produced a combined likelihood ratio of 300 million. SNP profiles produced a combined likelihood ratio of 3.34 E46, assuming independence among the SNPs. These likelihood ratios strongly supported this pedigree structure. Population background information generated by the Ancestry SNP panel also supported this pedigree structure.

### Concordance with data provided at end of blinded study

**Figure 5:**
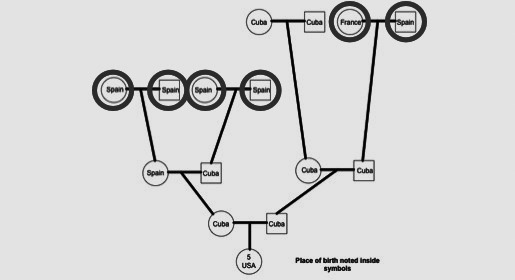
Results with apparent discordance at sample five that eventually were supported with additional pedigree meta data.

After completing the genetic analyses, the sample providers supplied all sample source data that had been collected on these 12 individuals, which then were compared with the genetic data generated in this blind study (Table 5). While primarily concordant, the two data sets exhibited a few discrepancies (Table 5), and in an effort to understand these discrepancies, additional information was requested from the sample providers. As an example, the donor of sample 5 self-reported as being from a Central Caribbean population, while the genetic data supported that she fit better with a European background. Consistent with the genetic data, this individual’s self-declared phenotype (brown hair, hazel eyes, white fair skin complexion) and extended pedigree (maternal great-grandparents are from Spain and paternal great-grandparents are from Spain, France, and Cuba) supported a European background (Figure 5). These overall results demonstrated the robustness of the genetic data that were generated.

**Table 5:** Population affiliation based on genetic data and that provided by Green Mountain sample providers (GM) at completion of blinded study

#	Maternal lineage	GM	Paternal lineage	GM	Biogeographic ancestry	GM
1	European	Western Europe	West Asia, Russian Plain, or Central Asia	American/Western Europe	European	Caucasian American
3	European	Southern Europe	Unknown	Southern Europe	European	Caucasian Southern Europe
4	European*	Southern Asia*	Central Asia, the Indian Subcontinent, Siberia	Southern Asia	Asian	Southern Asia
5	European*	Central Caribbean*	Unknown	Central Caribbean	European*	Central Caribbean*
6	European	Southern Europe	Arabian Peninsula	Southern Europe	European	Southern Europe
7	Asian	Eastern Asia	Asia	Eastern Asia	Asian	Eastern Asia
10	European	Eastern Europe	Unknown	Caucasian American	European	Caucasian American/Eastern Europe
13	African	African American	Unknown	African American	African American	African American
14	European	Eastern European	Unknown	Central Caribbean	African admix	American/Central Caribbean
15	European	Central Caribbean/Eastern European	Unknown	South American	African admix	South American/Central Caribbean
16	African	Central Caribbean/African	Africa	Central Caribbean/African	African	Central Caribbean/African
17	European	Eastern European	Unknown	Eastern European	European	Eastern European

*Indicates slight discrepancies found in population background assignments supported by genetic data and population background initially supplied by the GM sample providers.

## Discussion

This blinded genetic study evaluated the utility of the PGM™ system in sequencing and analyzing large, forensically relevant genetic marker panels. Four genetic marker systems were run manually on the PGM™ in about 1 week’s time by 1 individual. Information obtained from typing these markers is useful in delineating the identity, sex, population ancestry, and familial relationships of unknown persons. Completeness of genetic profiles, depth of coverage, strand balance, and allele balance were evaluated as informative metrics for the quality and reliability of the data produced, and each metric indicated that thorough and accurate data had been generated. Additionally, all results from analysis of the 12 genomic samples were consistent with sample source information provided by sample providers at the end of the blinded study. While limited to only 12 individuals, this study demonstrated the feasibility of typing a large multiplex of forensically relevant genetic markers on the PGM™ system.

Full profiles were obtained for all 12 samples with SNP, STR, and mitochondrial DNA marker systems. Only 1 ng of DNA was used to prepare each library, and results showed there was consistently high coverage across all markers analyzed. Successful typing at 1 ng of input DNA and relatively high coverage suggest that this MPS technology has the potential sensitivity of detection that could reach the same level of current DNA typing technologies. While there was little variation in coverage among samples, there was some variation in coverage among the different markers. These differences in coverage from marker to marker are likely due primarily to differences in PCR amplification efficiency. However, the dynamic range for varied coverage is accommodated better with MPS systems than with CE-based systems. With the latter methodology, a multiplex marker system with some markers having very high signal output can impact substantially the signal of low signal markers. High and low signal markers with MPS data can be tolerated without distortion of signal output. The differences in coverage seen in autosomal SNPs vs Y-SNPs in the Identity Panel likely can be attributed to the additional coverage the autosomal SNPs receive in females due to the lack of a Y-chromosome. This trend also can be seen in the differences in autosomal SNP coverage between males and females. For example, rs1490413, an autosomal SNP in the Identity Panel, had an average coverage of 2385 × ± (613) in males vs an average coverage of 2703 × (±675) in females. Strand balance calculations showed that coverage was obtained from both strands of the DNA for each marker set, reinforcing the accuracy of the genotype calls. ACRs displayed well-balanced data for all the markers analyzed indicating that the risk of allele drop-out was low for the amount of input DNA used in the study. The complete profiles, coverage, strand balance, and allele coverage ratios seen in this study support robust and reliable data.

In this study, population ancestry assignments were limited to major populations. This process is marker dependent, reference population dependent, and software dependent. Limiting the assignments to major populations allowed for higher confidence in assignments. Despite these efforts, slight discrepancies were found in the population background assignments supported by genetic data and the population background reported by the donors of the samples (Table 5). As previously stated, further evaluation of the individual’s self-declared phenotype and pedigree helped resolve these discrepancies. However, in studies such as these, care must be taken to recognize that biogeography does not always equate to bioancestry. Additionally, admixed individuals and population assignment is a more complex process. As technology continues to improve and the amount of available sequence data on reference populations continues to grow, the ability to genetically predict the bioancestry of an unknown person should improve as well.

MPS offers an avenue for expanding forensic typing capabilities. When analyzing STRs, some loci displayed within repeat sequence variants that cannot be detected by CE methods. This additional variation allows for greater discrimination power for such loci, which is helpful when trying to differentiate between individuals with similar profiles at those loci. In addition, sequence variants within STRs may help resolve mixtures better than can be accomplished with CE data. For example, several individuals in this study had the same size allele 13 at the D8S1179 locus (Table 4). Consider if samples 1 and 14 were mixed, sequence level information would help with interpretation at this locus due to the varying SNPs located in these individuals’ alleles. Finally, these intra repeat sequence variants can aid in familial analysis of unknown individuals. Consider the pedigree identified in this study and their genotypes at the D8S1179 marker: sequence variants present among the alleles at this marker allow for determination of exactly which 13 allele Sample 14 inherited from each parent and which of those alleles she passed along to her own offspring.

The results of this blinded study indicate that the PGM™ is a promising platform for forensic genetics analyses. The results also support that the MPS platform, chemistries, and software used are sufficiently reliable to continue and perform full validation studies, which already are under way. Validation efforts will focus on sensitivity of detection, expanding population data sets, determining stochastic effect levels, evaluating reproducibility, and analyzing mixtures and mock case samples.
